# Retinal Vascular Occlusion after Severe Acute Respiratory Syndrome Coronavirus Vaccination: A Vaccine Adverse Events Reporting System Database Analysis

**DOI:** 10.1016/j.xops.2023.100354

**Published:** 2023-06-20

**Authors:** Rohan Bir Singh, Uday Pratap Singh Parmar, Rudraksh Gupta, Antonio Jacobo Vega Garcia, Wonkyung Cho, Kanwar Partap Singh, Aniruddha Agarwal

**Affiliations:** 1 Massachusetts Eye and Ear, Harvard Medical School, Boston, Massachusetts; 2 Department of Ophthalmology, Leiden University Medical Center, Leiden, the Netherlands; 3 Discipline of Ophthalmology and Visual Sciences, Faculty of Health and Medical Sciences, Adelaide Medical School, University of Adelaide, Australia; 4 Department of Ophthalmology, Government Medical College and Hospital, Chandigarh, India; 5 Department of Radiology, Yale School of Medicine, New Haven, Connecticut; 6 Eye Institute, Cleveland Clinic Abu Dhabi, Abu Dhabi, United Arab Emirates; 7 Cleveland Clinic Lerner College of Medicine, Case Western Reserve University, Cleveland, Ohio; 8 Department of Ophthalmology, Maastricht University Medical Center+, Maastricht, the Netherlands

**Keywords:** Retinal artery occlusion, Retinal vein occlusion, Retinal vessel occlusion, SARS-CoV-2

## Abstract

**Purpose:**

To evaluate the cases of retinal artery occlusion (RAO) and retinal vein occlusion (RVO) after severe acute respiratory syndrome coronavirus (SARS-CoV-2) disease 2019 vaccination.

**Design:**

Retrospective study of the cases reported to the Centers for Disease Control and Prevention Vaccine Adverse Events Reporting System between December 11, 2020 and July 1, 2022.

**Participants:**

Patients diagnosed with RVO after vaccination with BNT162b2, mRNA-1273, and Ad26.COV2.S, globally.

**Methods:**

We performed a descriptive analysis of the demographics and presentation in patients with RVO. The correlations between the vaccines and continuous and categorical variables were assessed. We performed the post hoc analysis to evaluate the association between RAO, RVO onset postvaccination, and vaccine and dosage. A 30-day reverse Kaplan-Meier analysis was conducted for RAO and RVO onset after vaccination.

**Main Outcome Measures:**

The crude reporting rate of RVO after SARS-CoV-2 vaccine. The ocular and systemic presentations, onset duration, and short-term risk of RAO and RVO after vaccination.

**Results:**

One thousand three hundred and fifty-one RVO cases were reported globally. The crude reporting rates for BNT162b2, mRNA-1273, and Ad26.COV2.S were 0.36, 0.41, and 0.69, respectively. The majority of the cases were reported after BNT162b2 (n = 606, 74.17%). The mean age of patients with RVO and RAO was 58.54 ± 16.06 years and 64.63 ± 16.16 years, respectively. Most cases of RVO (41.12%) and RAO (48.27%) were reported within the first week. The mean onset interval for RVO was significantly longer in patients who received Ad26.Cov2.S (54.07 ± 88.98 days) compared with BNT162b2 (18.07 ± 28.66 days) and mRNA-1273 (22.85 ± 38.13 days) vaccines (*P* < 0.0001). This was confirmed by post hoc analysis (*P* < 0.0001). The reverse Kaplan-Meier 30-day risk analysis showed a significant a higher risk of RVO onset after BNT162b2 compared with other vaccines (*P* < 0.0001).

**Conclusions:**

The low crude reporting rate highlights a low safety concern for RVO after SARS-CoV-2 vaccination. This study provides insights into possible temporal association between reported RVO events with SARS-CoV-2 vaccines; however, further insights are needed to understand the underlying immunopathologic mechanisms that promote thrombosis of retinal vasculature on vaccine administration.

**Financial Disclosure(s):**

The author(s) have no proprietary or commercial interest in any materials discussed in this article.

Since March 2020, the global pandemic outbreak caused by the rapidly spreading severe acute respiratory syndrome coronavirus (SARS-CoV-2) has led to severe morbidity and mortality. The global immunization efforts deployed against the causative virus have been an effective preventative and control to mitigate the impact of this global pandemic. The vaccines were swiftly developed and received emergency use authorization (EUA) in the months after the pandemic outbreak. In the United States, 3 vaccines received EUA from the United States Food and Drug Administration (FDA): BNT162b2 (Pfizer Inc/BioNTech SE), Ad26.Cov2.S (Janssen Pharmaceuticals/Johnson & Johnson), and mRNA-1273 (Moderna Inc).^1^^,^^2^ After FDA EUA, these vaccines received the requisite regulatory approval in several other countries.^3^, ^4^, ^5^

Since the initiation of the worldwide vaccination efforts, several ophthalmic adverse events have been reported in the literature.^6^, ^7^, ^8^, ^9^, ^10^, ^11^, ^12^, ^13^ The Centers for Disease Control and Prevention (CDC) included the SARS-CoV-2 vaccine-associated adverse event reporting in the Vaccine Adverse Effect Reporting System (VAERS). In 1990, CDC established the VAERS database for passive surveillance of FDA-approved vaccines as a warning system assessing potential adverse events after vaccination.^14^ The database records adverse event data and records local and systemic side effects. These include ocular disorders, such as retinal vessel occlusion, as potential adverse effects after vaccination. The published literature assessing the association between retinal vessel occlusion and SARS-CoV-2 vaccination is limited to case reports, series, and review articles.^15^, ^16^, ^17^, ^18^, ^19^, ^20^, ^21^, ^22^, ^23^, ^24^, ^25^, ^26^, ^27^, ^28^, ^29^, ^30^, ^31^, ^32^, ^33^, ^34^, ^35^^,^ Although the evidence in the literature speculated a causal association between the adenoviral vector and mRNA-based SARS-CoV-2 vaccines, the precise mechanism is not completely understood. An immunologic basis has been hypothesized, ultimately leading to the activation of platelets causing dysregulated coagulation.^36^ Autoantibodies against the platelet factor 4, direct binding of the naked DNA or RNA to the platelets causing macromolecular complex formation, the inherent affinity of the adenovirus for platelets, and vaccination-induced systemic inflammation and cytokine upregulation and tissue factor activation have been speculated as potential triggers for this downstream cascade.^37^^,^^38^ To better understand the temporal association between retinal vessel occlusion and SARS-CoV-2 vaccinations and presenting clinical features in these patients, we evaluated the largest vaccine-associated retinal vessel occlusion case cohort recorded in the CDC-VAERS database. In this study, we assessed the crude reporting rate (CRR) of retinal vessel occlusion cases since the inception of the vaccination efforts in December 2020. We also evaluated the clinical presentations in patients with retinal vessel occlusion after vaccination and association between patient demographics and adverse event onset duration after vaccination.

## Methods

### Data Source

This database analysis was conducted using the publicly available database provided by VAERS (CDC). The VAERS database was established 3 decades ago to monitor vaccine safety by reporting incidence of known short- and long-term adverse events after vaccination, detecting atypical and rare vaccine-associated adverse events, and identifying the risk factors causing these events. The database consists of reports from patients, parents (for minor patients), clinicians, vaccine manufacturers, and regulatory bodies from all over the world. The patient information in the reports is deidentified and anonymized before being added to the database.

The adverse events reports submitted to VAERS are published by the Wide-ranging Online Data for Epidemiologic Research platform. The CDC developed and currently operates this platform.^39^ The adverse event reports include patient demographic data, vaccine administration and adverse event onset date, detailed adverse event history, clinical history, current medications and comorbidities, and unstructured report of clinical presentation and adverse event diagnosis after administration of the vaccine. To ensure the integrity and accuracy of the reports, the submissions are reviewed by the CDC and FDA. The false reports violate United States federal law under 18 U.S. Code § 1001 and are punishable by imprisonment and fine. Subsequently, the reports are assessed by a professional third party for assigning appropriate medical terminology as per Medical Dictionary for Regulatory Activities terms.^40^ On requesting the CDC for permission to analyse and publish these data, we were informed that this database can be accessed freely and no further permission was required to use, copy, distribute, and publish these data analyses.^41^ We conducted this study in compliance with the National Statement on Ethical Conduct in Human Research and tenets of the Declaration of Helsinki. The data included in this study are publicly available, deidentified, and anonymous, therefore, the University of Adelaide Human Research Ethics Committee exempted it from review.

### Patient Cohort

The patients with retinal vessel occlusion included in this retrospective study were diagnosed with retinal artery and vein occlusion after receiving mRNA-1273, Ad26.Cov2.S, and BNT162b2 vaccines between December 11, 2020 and July 1, 2022. The data were grouped by age, sex, location, symptoms, and onset interval postvaccination. Additionally, the adverse event data included clinical presentation, laboratory reports, adverse events after previous vaccinations, current medications, and allergies. The patient location was assessed using the standardized international organization of standardization (ISO) code assigned in the Wide-ranging Online Data for Epidemiologic Research platform. Four authors (R.B.S., U.P.S., R.G., and A.J.V.G.) manually extracted the data points of interest from the unstructured adverse event description for further analysis.

### Statistical Analysis

The data were analyzed using R Studio (R Foundation for Statistical Computing). We estimated the CRR of retinal vessel occlusion per million doses for the 3 SARS-CoV-2 vaccines. Furthermore, a descriptive analysis of the demographic and vaccination data was performed. The association between the retinal vessel occlusion onset interval after vaccination and type of vaccine, age, sex, and dosage were evaluated using the 1-way analysis of variance test. Furthermore, we performed the post hoc analysis to assess the association between onset interval between age groups, number of doses, and vaccine type. Finally, reverse Kaplan-Meier analysis was also performed for a comparative analysis of retinal artery and retinal vein occlusion (RVO) between the different vaccines. The missing data values in the reports were accounted for using the Na.rm code during analysis. The *P* values of < 0.05 were considered statistically significant.

## Results

During the study period, 2 061 557 270 SARS-CoV-2 vaccine doses including BNT162b2 (80.7%), mRNA-1273 (16.8%), and Ad26.COV2.S (2.5%) were administered. A total of 1 250 310 (0.06% of all doses) adverse events were reported after coronavirus disease 2019 (COVID-19) vaccination, including 1351 retinal vessel occlusion cases. Among these cases, 817 patients (60.5%) were diagnosed with RVO, 433 cases (32.05%) were diagnosed with retinal arterial occlusion (RAO), and 101 cases (7.47%) were unclassified cases. The unclassified cases were excluded from further analyses. The cases of RAO and RVO after administration of SARS-CoV-2 vaccine were reported from Europe (64.9%, 44.1%), North America (18.0%, 27.9%), Asia (3.9%, 4.9%), Australia (1.6%, 1.4%), Africa, and South America (0.1%, 0.2%). The patients diagnosed with RVO had received BNT162b2 (606, 74.17%), mRNA-1273 (143, 17.50%), and Ad26.COV2.S (36, 4.41%). Among the patients diagnosed with RAO, 324 patients (74.83%), 83 patients (19.17%), and 23 patients (5.31%) had received BNT162b2, mRNA-1273, and Ad26.COV2.S, respectively.

The CRRs (per million doses) of retinal vessel occlusion for BNT162b2, mRNA-1273, and Ad26.COV2.S were 0.36, 0.41, and 0.69, respectively. A comparable CRR for RAO was observed after administration of BNT162b2 (0.19), mRNA-1273 (0.24), and Ad26.COV2.S (0.45) vaccines. Mean age at presentation for RVO was 58.54 ± 16.06 years and RAO was 64.63 ± 16.16 years (Table 1). A comparable proportion of patients with RVO (48.47%) and RAO (47.81%) were female. A significant correlation was observed between the age of patients and incidence of RVO (*P* = 0.0412) and RAO (*P* = 0.0031). We observed that 70.6% of RVO cases were reported in patients aged 41 to 80 years and 84.5% of RAO cases were reported in patients in the same age groups. A significantly higher proportion of the RVO (*P* = 0.0021) and RAO (*P* = 0.0317) cases were reported after BNT162b2 (RVO, 74.17%; RAO, 74.8%) compared with mRNA1273 (RVO, 17.50%; RAO, 19.17%) and Ad26.CoV2.S (RVO, 4.41%; RAO, 5.31%). However, because the CRR was lowest for BNT162b2, this difference is primarily due to the higher number of doses administered of this vaccine.

**Table 1:** The Demographics and Vaccination Data of Patients who were Diagnosed with Retinal Vascular Occlusion after SARS-CoV-2 Vaccination

	Retinal Venous Occlusion			Retinal Arterial Occlusion		
No. of cases reported	817		*P* value	433		*P* value
Mean age (yrs)	58.54 ± 16.06			64.63 ± 16.16		
Age						
0–10	0	0	0.0412∗	0	0.00	0.0031∗
11–20	7	0.86%		3	0.69%	
21–30	32	3.92%		14	3.23%	
31–40	73	8.94%		21	4.85%	
41–50	108	13.22%		36	8.31%	
51–60	182	22.28%		68	15.70%	
61–70	158	19.34%		105	24.25%	
71–80	129	15.79%		95	21.94%	
81–90	56	6.85%		62	14.32%	
91–100	6	0.73%		6	1.39%	
Unknown	66	8.08%		23	5.31%	
Sex						
Female	396	48.47%	0.6372	207	47.81%	0.6121
Male	379	46.39%		216	49.88%	
Unknown	42	5.14%		10	2.31%	
Origin						
Europe	530	64.87 %	0.0712	191	44.11 %	0.1241
North America	147	17.99 %		121	27.94 %	
Asia	32	3.92 %		21	4.85 %	
South America	1	0.12 %		1	0.23 %	
Australasia	13	1.59 %		6	1.39 %	
Africa	1	0.12 %		1	0.23 %	
Unknown	93	11.38 %		92	21.25 %	
Type of vaccine			0.0021∗			0.0317∗
BNT162b2	606	74.17%		324	74.83%	
mRNA-1273	143	17.50%		83	19.17%	
Ad26.Cov2.s	36	4.41%		23	5.31%	
Unknown	32	3.92%		4	0.92%	
Dosage			0.422			0.3816
1	285	34.88%		161	37.18%	
2	291	35.62%		152	35.10%	
3	94	11.51%		42	9.70%	
4	3	0.37%		2	0.46%	
Unknown	144	17.63%		76	17.55%	
Onset interval postvaccination			0.2731			0.3112
Day 0	106	12.97%		59	13.63%	
Days 1–7	230	28.15%		150	34.64%	
Days 8–14	106	12.97%		42	9.70%	
Days 15–21	81	9.91%		41	9.47%	
Days 22–28	37	4.53%		16	3.70%	
Days > 28	141	17.26%		90	20.79%	
Unknown	116	14.20%		35	8.08%	

SARS-CoV-2 = severe acute respiratory syndrome coronavirus.

∗ Indicates statistically significant at *P* < 0.05.

Among the patients diagnosed with RVO, 285 (34.88%), 291 (35.62%), 94 (11.5%), and 3 (0.4%) cases occurred after the first, second, third, and fourth doses of vaccine, respectively (Table 1). Retinal arterial occlusion was diagnosed in 161 cases (37.2%) after first dose, 152 cases (35.1%) after second dose, 42 (9.7%) after third dose, and 2 cases (0.5%) after third dose. The majority of the RVO cases were reported within 1 week of the vaccine administration, including 442 patients (54.1%) diagnosed with RAO and 251 (58.0%) patients diagnosed with RVO. Interestingly, 13% of the RAO and RVO cases were reported on the day of the vaccination.

We observed a significantly shorter RVO onset interval (*P* < 0.001) in patients who received BNT162b2 (18.07 ± 28.66 days) compared with mRNA-1273 (22.85 ± 38.13 days) and Ad26.Cov2.S (54.07 ± 88.98 days). Interestingly, the onset intervals were comparable for RAO onset for the 3 vaccine types (Table 2). On performing the post hoc analysis, we observed a significant difference only between the onset interval of BNT162b2 compared with Ad26.Cov2.s (*P* < 0.001), whereas it was comparable with mRNA-1273 (*P* = 0.5057). Retinal vein occlusion onset duration had a significant association with age (*P* = 0.006) and the shortest onset duration was observed in patients in their fifth decade (14.11 ± 16.18 days). Interestingly, the onset duration in patients aged 21 to 30 years was significantly longer (60.03 ± 90.01 days) than all other age groups (Table 2). On the contrary, the RAO onset durations were comparable for all age groups with the shortest observed in patients in their eighth decade (15.47 ± 31.35 days). Additionally, the RVO and RAO onset intervals were comparable in men and women.

**Table 2:** Analysis to Assess the Factors Associated with Onset Interval of the Retinal Vessel Occlusion after SARS-CoV-2 Vaccination

	Retinal Vein Occlusion		Retinal Artery Occlusion	
	Mean Onset Interval (in Days)	*P* Value	Mean Onset Interval (in Days)	*P* Value
Vaccine∗				
BNT162b2	18.07 ± 28.66	**< 0.001**	22.55 ± 40.00	0.723
mRNA-1273	22.85 ± 38.13		28.40 ± 50.82	
Ad26.Cov2.s	54.07 ± 88.98		27.17 ± 36.27	
Sex				
Female	18.65 ± 28.73	0.1653	23.21 ± 42.57	0.7591
Male	22.46 ± 41.85		24.51 ± 41.69	
Age				
0–10	0	**0.0056**	0	0.288
11–20	24.28 ± 37.94		27.33 ± 27.50	
21–30	60.03 ± 90.01		20 ± 20.17	
31–40	17.03 ± 23.69		38.61 ± 49.02	
41–50	14.11 ± 16.18		30.15 ± 54.17	
51–60	22.31 ± 38.06		30.69 ± 46.10	
61–70	16.6 ± 27.15		19.43 ± 37.44	
71–80	19.54 ± 30.77		15.47 ± 31.35	
81–90	20.96 ± 34.94		29.31 ± 51.15	
91–100	7.33 ± 6.02		50.2 ± 83.16	
Dosage				
1	19.01 ± 36.64	0.249	17.55 ± 33.21	0.422
2	25.66 ± 40.93		29.82 ± 49.80	
3	15.09 ± 23.65		29.78 ± 41.63	
4	2 ± 3.46		3 ± 4.24	

SARS-CoV-2 = severe acute respiratory syndrome coronavirus.

∗ One-way analysis of variance test and unpaired *t* test.

The comparative analysis of the risk factors, clinical history, and systemic presentation in patients diagnosed with retinal vessel occlusion after vaccination BNT162b2 and mRNA-1273 are summarized in Table 3. Because very few cases of retinal vessel occlusion were reported after Ad26.CoV2.S, this vaccine was excluded from this analyses. A history of hypertension (17.76%), stroke (7.52%), diabetes mellitus (7.12%), and smoking (6.48%) were the most common risk factor in patients who were diagnosed with retinal vessel occlusion after vaccination. A significantly higher proportion of reported retinal vessel occlusion cases after BNT162b2 (4.62%; *P* = 0.0369) had previously received intravitreal injections compared with mRNA-1273 (1.33%) recipients. These data highlight the possibility of a higher risk of vessel occlusion after BNT162b2 in patients with a previous history of retinal vascular disorders. Interestingly, a significantly (*P* < 0.001) higher proportion of retinal vessel occlusion cases who received mRNA-1273 (35.84%) vaccine compared with BNT162b2 (11.29%) had a history of COVID-19.

**Table 3:** Comparative Analysis of the Patients with History and Systematic Presentation in Patients with Retinal Vessel Occlusion after SARS-CoV-2 Vaccination

	BNT162b2	mRNA-1273	*P* Value	Chi-square
Risk factors and clinical history				
Hypertension	185 (19.98%)	37 (16.37%)	0.2636	1.249
Diabetes mellitus	66 (7.10%)	23 (10.18%)	0.1573	2.003
Hypercholesterolemia	17 (1.83%)	4 (1.77%)	1	< 0.0001
Intravitreal injections	43 (4.62%)	3 (1.33%)	**0.0369**	4.3531
Stroke	72 (7.74%)	22 (9.73%)	0.3993	0.7103
Ischemic heart disease	23 (2.47%)	5 (2.21%)	1	< 0.0001
Smoking	68 (7.31%)	13 (5.75%)	0.495	0.4656
Glaucoma	33 (3.55%)	6 (2.65%)	0.6423	0.2157
Arteritis	11 (1.18%)	3 (1.33%)	1	< 0.0001
Systemic lupus erythematosus	25 (2.69%)	1 (0.44%)	0.1607	1.4212
Systemic vasculitis	24 (2.58%)	2 (0.88%)	0.1958	1.6738
COVID-19	105 (11.29%)	81 (35.84%)	**< 0.001**	79.207
Systemic presentations				
Fever	23 (2.47%)	9 (3.98%)	0.3117	1.0233
Lymphadenopathy	6 (0.65%)	0	0.4868	0.4836
Malaise	15 (1.61%)	2 (0.88%)	0.6108	0.25907
Vomiting	3 (0.32%)	0	0.8991	0.01607
Chest pain	7 (0.75%)	2 (0.88%)	1	< 0.001
Hypersensitivity	7 (0.75%)	0	0.4059	0.6907

COVID-19 = coronavirus disease 2019; SARS-CoV-2 = severe acute respiratory syndrome coronavirus.

Central artery and vein occlusions were observed in 148 (11.48%) patients, whereas the remaining cases had branched vessel occlusions. A significantly higher proportion of patients who received the BNT162b2 vaccine (13.98%) presented with central occlusion compared with those who received mRNA-1273 vaccine (7.96%; *P* = 0.02). Macular edema was the most common (10.72%) ocular presentation in patients after vaccination. The other ocular presentations included retinal vasculitis, hemorrhages, detachment, cotton wool spots, and papilledema (Table 4). The medications prescribed to the patients at presentation are outlined in Table 4. A significantly higher number of patients who received BNT162b2 vaccine were diagnosed with retinal vessel occlusion despite being on anticoagulants (92 vs. 1; *P* = 0.0014) and statins (n = 71 vs. 5; *P* = 0.005) compared with those who received the mRNA-1273 vaccine.

**Table 4:** The Ocular Presentations in Patients with Retinal Vessel Occlusion after SARS-CoV-2 Vaccination

	BNT162b2	mRNA-1273	*P* Value	Chi-square
Ocular presentations				
Macular edema	116 (12.47%)	18 (7.96%)	0.0737	3.1968
Retinal vasculitis	24 (2.58%)	2 (0.88%)	0.1958	1.6738
Retinal hemorrhages	45 (4.84%)	8 (3.54%)	0.5073	0.4395
Cilioretinal artery occlusion	8 (0.86%)	0	0.3407	0.90775
Cotton wool spots	3 (0.32%)	0	0.8991	0.01607
Retinal detachment	9 (0.97%)	1 (0.44%)	0.7146	0.1336
Papilledema	5 (0.54%)	3 (1.33%)	0.4032	0.6986
Medications				
Steroids	10 (1.08%)	3 (1.33%)	1	< 0.0001
Insulin	10 (1.08%)	2 (0.88%)	1	< 0.0001
Anticoagulants	92 (11.26%)	1 (0.44%)	**0.0014**	10.146
Aspirin	35 (3.76%)	7 (3.10%)	0.776	0.0809
Statins	71 (7.63%)	5 (2.21%)	**0.005**	7.858

SARS-CoV-2 = severe acute respiratory syndrome coronavirus.

**Figure 1:**
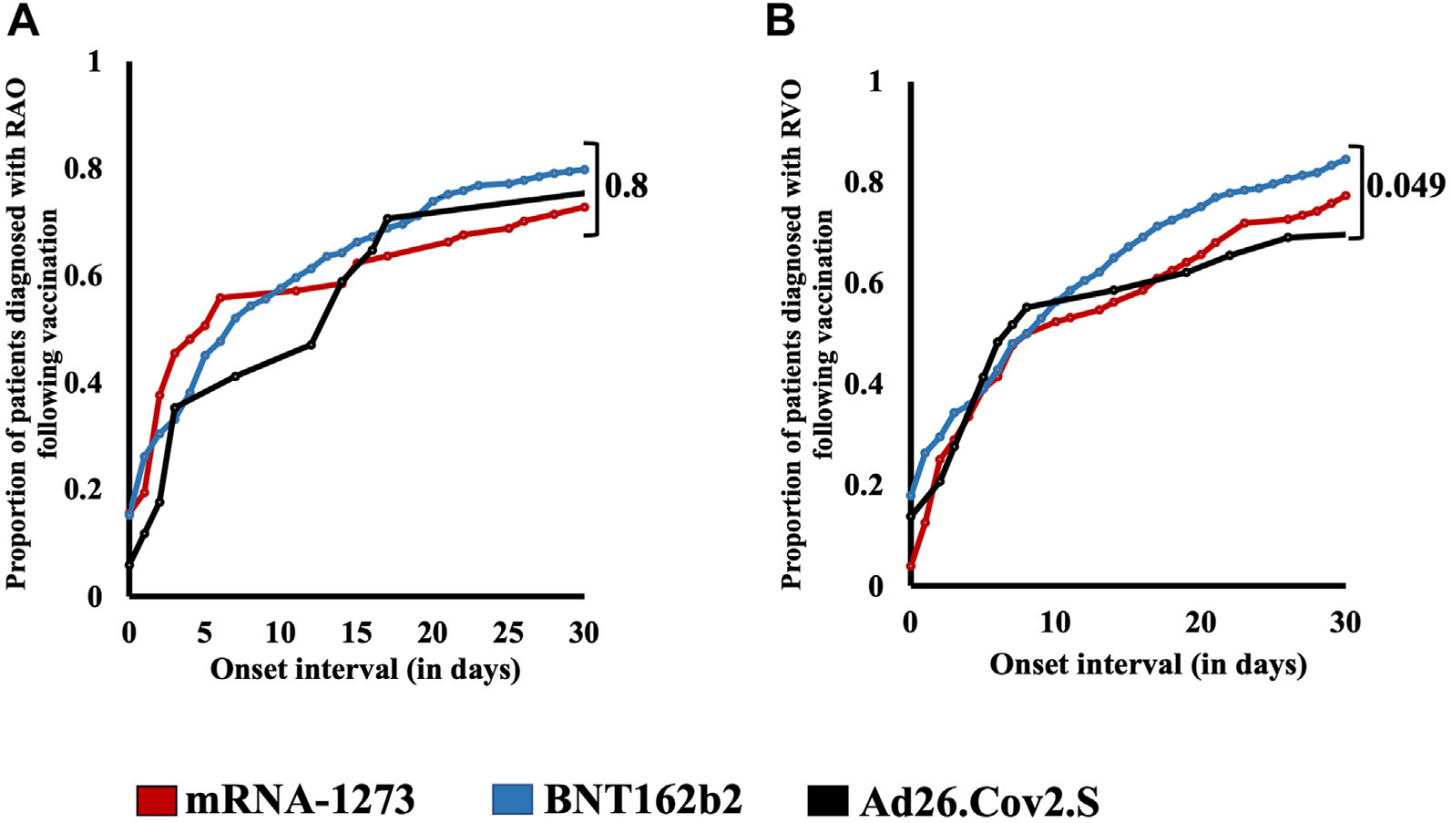
The 30-day reverse Kaplan-Meier analysis for (**A**) retinal artery occlusion (RAO) and (**B**) retinal vein occlusion (RVO) after vaccination with mRNA-1273, BNT162b2, and Ad26.CoV2.S cases.

The 30-day reverse Kaplan-Meier survival analysis revealed the risk of RAO after the 3 vaccines was comparable (*P* = 0.8; Fig 1A). The 30-day risk of RVO incidence was significantly higher after BNT162b2 vaccination in comparison mRNA-1273 and Ad26.Cov2.S vaccine (*P* = 0.049; Fig 1B). The post hoc analysis showed that RVO onset interval was significantly longer in patients who received Ad26.CoV2.S compared with BNT162b2 and mRNA-1273 (Table 5a). We did not observe an association between the onset RAO and RVO interval and vaccine dosage (Table 5b).

**Table 5:** Post Hoc Analysis Comparing Onset Interval Between (a) Vaccines and (b) Doses

Retinal Venous Occlusion	BNT162b2	mRNA-1273	Ad26.Cov2.s
BNT162b2	1		
mRNA-1273	0.5057		
Ad26.Cov2.s	< 0.0001	< 0.0001	1
Retinal arterial occlusion			
BNT162b2	1		
mRNA-1273	0.6976	1	
Ad26.Cov2.s	0.9995	0.9714	1

Retinal Venous Occlusion	First Dose	Second Dose	Third Dose	Fourth Dose
First dose	1			
Second dose	0.2056	1		
Third dose	0.9013	0.1197	1	
Fourth dose	0.9230	0.7816	0.9706	1
Retinal arterial occlusion				
First dose	1			
Second dose	0.0918	1		
Third dose	0.4604	1	1	
Fourth dose	0.9884	0.8971	0.9031	1

## Discussion

The global vaccination program against SARS-CoV-2 was essential for mitigating the extensive morbidity and mortality that was associated with the spread of the virus. In the United States, the 3 FDA-approved vaccines—BNT162b2, mRNA-1273, and AD26.COV2.S—have stemmed the impact of rapidly spreading virus by reducing disease severity, hospitalization, and chronic effects of COVID-19.^42^^,^^43^ The BNT162b2 and mRNA-1273 consist of modified mRNA which is translated into membrane anchored, SARS-CoV-19 spike proteins. The recognition of these proteins by the body’s immune system induces a humoral and cellular immune response in the recipients, conferring immunity against the virus.^44^^,^^45^ In contrast, Ad26.Cov2.S vaccine induces immunity against the virus by a replication-incompetent, recombinant adenovirus type 26 (Ad26) viral vector expressing the SARS-CoV-2 spike protein in a stabilized conformation.^3^, ^4^, ^5^

The vaccines were developed in record time and received subsequent EUA, therefore, the data on their potential short and long-term, local, and systemic adverse effects, including ocular disorders, are limited. Several cases of vaccination-associated thrombotic events have been reported in association with the viral-vectored vaccines (AZD1222/ChAdOx1 nCoV-19 and Ad26.COV2.S) and even led to a safety alert issued by the Royal College of Ophthalmologists in the United Kingdom.^46^ These reports include cases of cerebral venous sinus thrombosis, immune thrombotic thrombocytopenia, deep vein thrombosis, and splanchnic venous thrombosis. This phenomenon was initially termed as “vaccine-induced pro-thrombotic immune thrombocytopenia” or “vaccine-induced immune thrombotic thrombocytopenia.^47^ Recently, the CDC has renamed it as “thrombosis with thrombocytopenia syndrome”.^48^ However, the evidence regarding retinal vessel occlusion after SARS-CoV-2 vaccination is limited to a few case reports and series and further research is needed to understand the underlying pathologic mechanisms.

Although a causal association between retinal vessel occlusion and SARS-CoV-2 has still not been established, it is speculated that dysregulated coagulation is due to the interaction between the vaccine and the platelet factor 4 (PF4) on platelets, subsequently causing platelet activation. Greinacher et al^49^ suggested that this interaction is due to the action of the immunoglobulin antibodies, which are either induced by the cross linking of vaccine with PF4 or platelets or autoantibodies against PF4 due to the strong inflammatory stimulus of vaccination. Another possible trigger for these PF4-reactive autoantibodies could be the RNA in the vaccine. DNA and RNA are known to form multimolecular complexes with PF4 which induce autoantibodies against PF4.^50^

The higher rate of thrombotic event with the adenoviral vaccines has been associated with the inherent affinity of the adenovirus to bind to platelets, causing platelet activation.^51^^,^^52^ Another hypothesis about vaccine-induced vascular occlusion may be caused by postvaccination cellular immune response to systemic inflammation, causing the release of various chemokines and cytokines including interferon-γ and -λ, tumor necrosis factor-alpha, interleukin-12, interleukin-8, and interleukin-6.^53^, ^54^, ^55^ The cytokines can upregulate the tissue factor and the extrinsic coagulation cascade and downregulate tissue type plasminogen activator, leading to platelet activation and, hence, induce a procoagulant state. In the past, retinal vessel thrombosis after Hepatitis B vaccination has been reported.^56^ The retinal vessel occlusion in this study was attributed to similar immunologic mechanisms.

### Limitations

This retrospective assessing the retinal vessel occlusion cases reported to CDC-VAERS after COVID-19 vaccination has a few limitations. A major limitation is the lack of control group to assess the odds ratio of retinal vessel occlusive disease in patients who received the vaccine compared with the patients who were unvaccinated. Centers for Disease Control and Prevention-VAERS is a passive surveillance system for adverse events reports from patients, physicians, drug regulatory bodies, and pharmaceutical companies. Despite the mandatory requisite to report all the adverse events after CDC approved vaccinations, under and delayed reporting are quite common. Additionally, the submitted reports are incomplete and lack uniformly reported data in some cases.

The adverse event reporting after COVID-19 vaccination is limited to a few countries in North America, European Union, Australia, and Asia. Therefore, there are no adverse effects reports from the developing nations where a large proportion of the vaccine recipients reside. Additionally, there are no retinal vessel occlusion reports after vaccination with ChAdOx1 nCoV-19, ZyCoV-D, Sputnik, Covidecia, Sputnik, Sinopharm, Abdala, Soberna, Zifivax, and Novavax recorded in the database. Hence, the data for similar events after these vaccines could not be analyzed. As there is no unvaccinated control group, the relative risk analysis could not be performed for this study. This database analysis has only evaluated the temporal association between retinal vessel occlusion and FDA-approved COVID-19 vaccination and does not demonstrate the underlying immunopathologic relationship; further studies are required to evaluate it.

The low CRRs indicate that retinal vessel occlusion is a rare adverse event after SARS-CoV-2 vaccination. The analysis shows that retinal vessel occlusion after vaccination is more likely to develop in patients with underlying cardiovascular risk factors like hypertension, diabetes mellitus, and stroke. The majority of the retinal vessel occlusion cases were reported in the first week after vaccination and were associated with the BNT161b2 vaccine. Therefore, the benefits of SARS-CoV-2 vaccine still outweigh the risks; however, retina specialists should be aware of the possibility of vaccine-associated retinal vessel occlusion. Therefore, the patients with underlying risk factors should be closely followed up after vaccination.
